# Genome wide identification of *BjSWEET* gene family and drought response analysis of *BjSWEET12* and *BjSWEET17* genes in *Brassica juncea*

**DOI:** 10.1186/s12870-024-05815-w

**Published:** 2024-11-19

**Authors:** Shuangping Heng, Jingjuan He, Xinyu Zhu, Jiayu Cai, Mengke Fu, Shaoheng Zhang, Wei Zeng, Feng Xing, Guangzhi Mao

**Affiliations:** College of Life Science, Institute for Conservation and Utilization of Agro-Bioresources in Dabie Mountains, Xinyang Normal University, Xinyang, 464000 P. R. China

**Keywords:** *B. juncea*, *SWEET* gene, Sugar transporter protein, Abiotic stress, Expression profile

## Abstract

**Background:**

Sugars Will Eventually be Exported Transporter (SWEET) gene family is a unique type of sugar transporter that plays a vital role in metabolic regulation, growth, development, and stress response in multiple species. This study aimed to systematically identify the SWEET gene family members and detect the regulation of gene expression and their potential roles of the SWEET gene family in *Brassica juncea*.

**Results:**

A total of 66 *BjSWEET* (*Brassica juncea* Sugar Will Eventually be Exported Transporter) genes distributed across 17 chromosomes were identified. The gene structure and motifs were relatively conserved, with all members containing the MtN3/saliva domain. Phylogenetic analysis revealed that the SWEET gene family can be classified into four subfamilies (Clades I, II, III, and IV). Collinearity analysis revealed that there were 118 pairs of segment duplicates, indicating that some *BjSWEET* genes were obtained via segmental duplication. The promoter regions of the *BjSWEET* genes contained many plant hormone-related response elements, stress-related response elements, growth and development elements, and light-responsive regulatory elements. Furthermore, analysis of the expression profiles revealed that the expression levels of the *BjSWEET* genes differed among the eight different tissues. qRT‒PCR analysis of six selected *BjSWEET* genes revealed that the expression levels of *BjSWEET17.2*, *BjSWEET17.4*, *BjSWEET12.2*, and *BjSWEET12.3* were significantly upregulated under drought treatment, suggesting that these genes may respond to drought stress in *B. juncea*.

**Conclusion:**

This study systematically identified and analyzed the SWEET gene family members in *B. juncea* for the first time, laying the foundation for further research on the molecular mechanisms of drought resistance in *B. juncea* and providing theoretical guidance for the application of these genes in other species.

**Supplementary Information:**

The online version contains supplementary material available at 10.1186/s12870-024-05815-w.

## Background

As the main carbon source and energy substances, soluble sugars such as glucose, fructose and galactose play essential roles in cytoskeleton formation, plant growth and development, energy metabolism, signal transduction, osmoregulation, and other processes [1, 2]. Sugar transporters are key mediators of sugar transport in plants, and sugars are transported from source to reservoir across the membrane, this process depends on sugar transporters. They can be widely found in fruits, veins, seeds, roots, pollen, and embryos [3]. To date, three main types of sugar transporters have been found in higher plants: SWEETs, monosaccharide transporters (MTs) and sucrose transporters (SUTs). Among them, SUT1 only exists in dicotyledonous plants, while SUT2 and SUT4 coexist in monocotyledonous and dicotyledonous plants, SUT3 and SUT5 only exist in monocotyledons. MTs are a protein superfamily, and gene expression can be regulated by environmental factors during cell development and can exhibit specific expression at different stages [4].

The SWEET gene family comprises bidirectional transmembrane transporters that can transport fructose, sucrose, and hexose in plants [5]. The typical SWEETs consist of seven transmembrane helices, which contain two tandem 3-TM repeat units, constituting the MtN3-slv domain or the PQ-loop domain, and the middle is connected by a less conserved transmembrane helix (TMH), thus comprising the TM SWEET structure of “3–1–3” [6]. In plants, the SWEET gene family can be classified into four subfamilies. The evolutionary sequences of the four subfamilies were as follows: Clade II, Clade I, Clade III and Clade IV [7]. Studies have shown that Clades I and II of the SWEET gene family mainly transport monosaccharides and preferentially transport hexose, while clade III transports disaccharides and mainly transports sucrose, Clade IV SWEETs function as fructose transporters in the vacuole [8]. To date, genome-wide analysis have identified SWEET gene family members in many plants, such as 17 in *Arabidopsis thaliana,* 21 in rice (*Oryza sativa*) [9], 52 in soybean (*Glycine max*) [10], 35 in potato (*Solanum tuberosum*) [11], 29 in tomato (*Solanum lycopersicum*) [12], and 23 in sorghum (*Sorghum bicolor*) [13]. Biochemical and functional analysis of the SWEET gene family revealed that SWEET proteins in plants were involved mainly in growth and development [14], host interactions, sugar compound distribution, transportation and storage, abiotic and biotic stress responses, and other expression regulatory processes [15]. For example, *AtSWEET13* can contribute to pollen development [16], *AtSWEET16* and *AtSWEET17* have been found to be involved in the regulation of root growth and development and the transport of vacuolar sugars in *Arabidopsis*, and *AtSWEET10* was reported to maintain cell viability in the *Arabidopsis* salt stress environment [17, 18]. *SlSWEET15* in *S. lycopersicum* was considered as an essential regulator of salt tolerance [19], and *SWEET9* in tobacco (*Nicotiana attenuata*) and Chinese cabbage (*Brassica rapa*) reportedly regulated nectar secretion [20].

Biotic and abiotic stresses affect plant growth and development and metabolic processes in many ways. In response to stress, plants alter their molecular structure and physiological and biochemical processes through a variety of signaling pathways. Under stress, soluble sugars play an important role in plants. Abiotic stress mainly includes drought, high temperature, low temperature, and ultraviolet radiation. Drought stress affects many physiological and biochemical processes in plants, including damage to the cell membrane, a decrease in the relative water content and chlorophyll content in leaves, and an increase in osmotic adjustment substances. Ultimately, plant performance is reduced in terms of yield and quality [21]. Therefore, studying the mechanism of plant growth under drought stress is important for *B. juncea* breeding. Previous studies have shown that *AtSWEET17* was transcriptionally upregulated under drought stress [22] and thus involved in the drought stress response in *Arabidopsis*. The *Arabidopsis* sucrose transporters SWEET11 and SWEET12 were phosphorylated under drought stress, and their gene expression increased significantly when the environment recovered, after which their expression remained constant [23].

With the development of genome sequencing technology, an increasing number of SWEET genes in *Brassicaceae* have been genome-wide identified. In *B. rapa*, after genome-wide analysis of the SWEET gene family, *BrSWEET* genes have been identified [24, 25]. *BoSWEET* genes in *B. oleracea* involved in response to chilling and clubroot disease [26]. A total of 68 members of the SWEET gene family have been identified in *B. napus*, and *BnSWEET12* was upregulated under drought stress [27]. *B. juncea* is an allotetraploid of *Brassica*, that is generated by spontaneous chromosome doubling after hybridization between two progenitor species, *Brassica nigra* and *Brassica rapa* [28]. It has been widely grown as an oilseed crop, economic vegetable, and condiment worldwide. However, genome-wide identification and analysis of the SWEET gene family have not been performed in *B. juncea*, and whether the *BjSWEET* genes participate in the drought stress response and the regulatory mechanism remain unknown. Therefore, understanding and studying the *SWEET* genes and their biological functions are essential for *Brassicaceae* breeding and cultivation of resistant varieties. In this study, we performed a genome-wide analysis of *SWEET* genes in *B. juncea* and the gene was renamed as *BjSWEET*. We subsequently analyzed the chromosome-based gene mapping, transmembrane helical structure, gene structure, conserved motifs, *cis*-acting elements in the promoters, phylogenetic relationships, and expression profiles of these genes. These findings lay a solid foundation for further elucidation of the biological function of *SWEET* genes in *B. juncea*.

## Results

### Identification and bioinformatics analysis of *BjSWEET* genes

A total of 66 SWEET gene family members were identified in *B. juncea* according to their respective orthologous genes in *Arabidopsis* (Table S1). These genes encode proteins with lengths ranging from 85 (BjSWEET8.4) to 411 aa (BjSWEET14.2), protein isoelectric points ranging from 4.77 to 9.72, and protein relative molecular weights between 9.68 (BjSWEET8.4) and 45.35 kDa (BjSWEET14.2). Approximately 92.42% of the BjSWEET proteins had isoelectric points higher than 7, indicating that most of the members were alkaline proteins. Subcellular localization prediction can help us to better explore molecular function. The results showed that BjSWEET8.3 was located in the nucleus, and that BjSWEET6.1 was located in the cell membrane and nucleus. BjSWEET8.4, BjSWEET8.5, BjSWEET10.1, BjSWEET10.2, and BjSWEET11.5 were localized in the cell membrane and chloroplasts. BjSWEET14.1 was localized in the cell membrane and peroxisomes. BjSWEET14.7 was localized in the cell membrane, chloroplast, Golgi and peroxisomes. BjSWEET17.2 was localized in the cell membrane and Golgi, while the remaining proteins were all localized in the cell membrane. These results indicated that BjSWEETs could perform biological functions in different subcellular organelles.

### Phylogenetic and multiple sequence alignment analysis of BjSWEETs

**Fig. 1:**
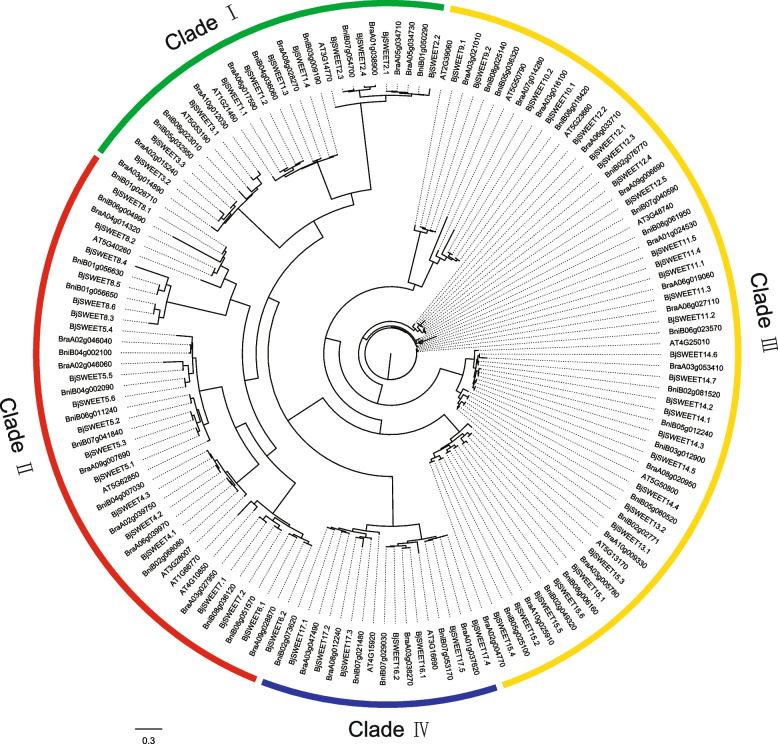
Phylogenetic tree of *B. juncea* SWEETs. The ML tree was constructed using MEGA6.06 software based on the SWEET protein sequences. Bootstrap values from 1000 replicates are displayed at each node. Four subfamilies were marked with different colors

To better understand the evolutionary relationships and functional implications of *BjSWEET* genes, we constructed the phylogenetic tree of *SWEET* genes from *B. juncea*, *B. rapa*, *B. nigra* and *A. thaliana* (Fig. 1). A total of 34 *SWEET* genes have been identified from *B. rapa* and 37 *SWEET* genes have been identified from *B. nigra*, respectively. The analysis revealed that the SWEET gene family members can be classified into four distinct subfamilies, namely Clades I-IV. Among them, Clade I had 11 *BjSWEETs*, 8 *BrSWEETs*, 6 *BniSWEETs*, and 3 *AtSWEETs* genes. Clade II had 19 *BjSWEETs*, 8 *BrSWEETs*, 12 *BniSWEETs* and 5 *AtSWEETs* genes. Clade III had 29 *BjSWEETs*, 14 *BrSWEETs*, 15 *BniSWEETs* and 7 *AtSWEETs* genes. Clade IV had 7 *BjSWEETs*, 4 *BrSWEETs*, 4 *BniSWEETs* and 2 *AtSWEETs* genes. The *SWEET* genes of the three *Brassica* species were clustered with their homologous genes from *A. thaliana*. Notably, Clades II and III contained a greater number of SWEET genes than Clades I and IV. And few *BjSWEETs* genes from Clade I, Clade II and Clade IV were lost during the evolution of *B. juncea*. The phylogenetic tree accurately depicted that the *SWEET* genes with close evolutionary relationships had little differentiation and high homology, suggesting that these genes may have functional redundancy. Multiple sequence alignment analysis of *BjSWEET* genes using MEGA 6.06 and GeneDoc software showed that all 66 *BjSWEET* genes had a segment of conserved sequence, constituting the conserved MtN3-slv or PQ-loop superfamily (Fig. S1). Furthermore, the conserved domain of the BjSWEET proteins was located at the N-terminus. This result was consistent with the localization of the conserved domain of the typical SWEET proteins.

### Gene structure, conserved motifs and transmembrane helices of BjSWEETs

**Fig. 2:**
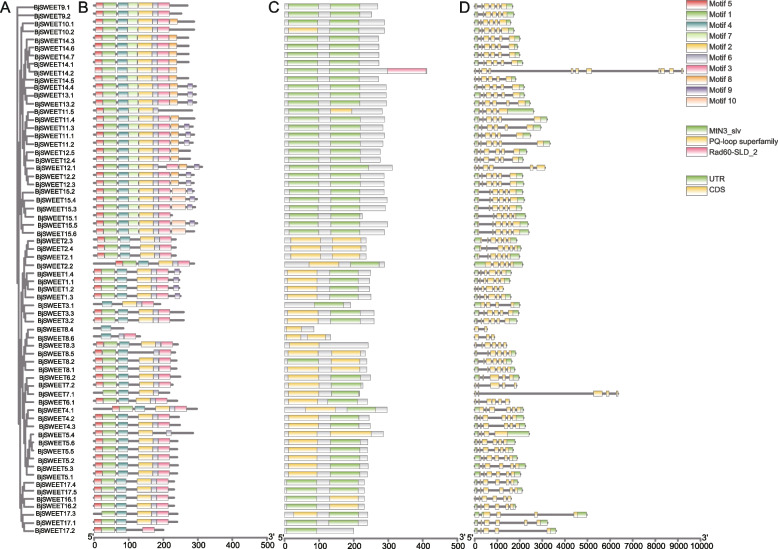
Detailed structure of SWEET family proteins in *B. juncea*. **A** Phylogenetic tree, (**B**) conserved motif, (**C**) conserved domain, (**D**) gene structure

**Fig. 3:**
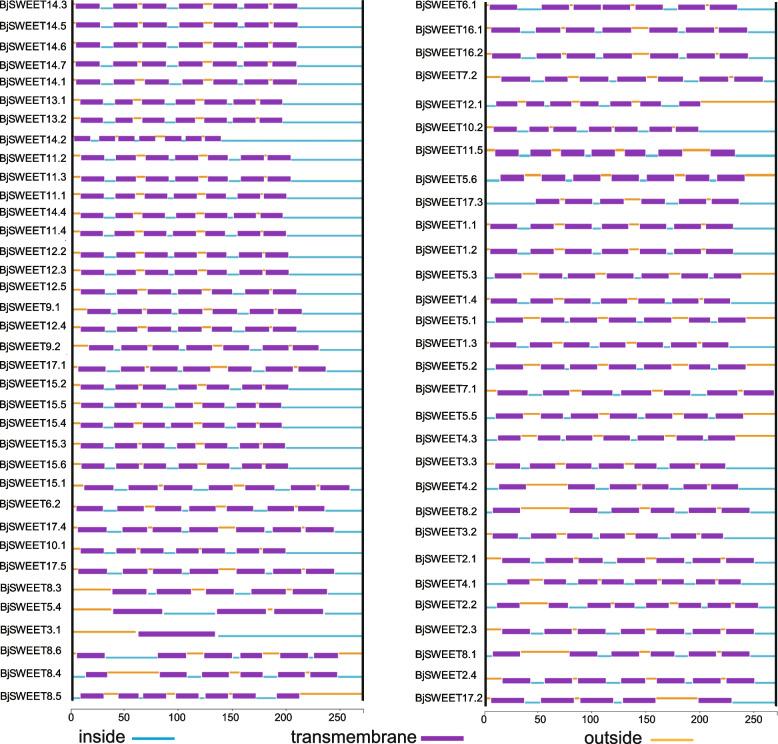
Transmembrane helices of SWEET family proteins in *B. juncea*. The whole sequence of each BjSWEET protein was labeled as inside transmembrane with blue color, transmembrane with purple color, and transmembrane outside with orange color

The BjSWEET proteins in the same subfamily had similar motifs, which was consistent with the results of the phylogenetic analysis. This indicated that these proteins may have similar functions (Fig. 2A). Conserved motifs can provide insights into the structural characteristics of BjSWEETs, and 10 conserved motifs were predicted (Fig. 2B, Table S2). The results found that all the BjSWEET proteins had 1 to 9 conserved motifs and all possessed motif 4, indicating that this motif is a unique SWEET domain of the SWEET gene family. Additionally, the same subfamily members contained similar conserved motifs, and certain motifs showed apparent specificity. For example, motif 7 and motif 8 were unique to Clade I (Fig. S2). However, *BjSWEET3.1*, *BjSWEET8.3*, *BjSWEET8.4* and *BjSWEET17.3* only possessed MtN3-slv domain or PQ-loop superfamily domain. This discrepancy may result in non-functionalization, sub-functionalization or neofunctionalization of these *SWEET* genes. Additionally, by analyzing the conserved domains of the SWEET gene family members, we observed that approximately 93.9% of *BjSWEETs* contained two domains, highlighting the essentiality of these domains for the normal expression of *SWEET* genes (Fig. 2C). The analysis of gene structure found that the number of introns of the *BjSWEET* genes differed (Fig. 2D). The *SWEET* genes in *B. juncea* exhibit structural diversity. The number of exons ranged from 2 to 11, and approximately 81.8% of the *BjSWEET* genes exhibited six exons. Most of the *BjSWEET* genes in Clade I subfamily had 6 exons expect *BjSWEET2.2* and *BjSWEET3.1*. The *BjSWEET* genes in Clade II subfamily varied greatly, only 2 exons have been found in *BjSWEET8.4*. Most of the *BjSWEET* genes in Clade III subfamily had 6 exons except *BjSWEET14.2* and *BjSWEET11.5*. The *BjSWEET17.3* gene had 4 exons, while other *BjSWEET* genes in Clade III subfamily had 6 exons. The analysis of transmembrane helical structures showed that the BjSWEET8.4 protein contained only 1 transmembrane helix (TMH), BjSWEET8.6 contained 3 TMHs, BjSWEET3.1 and BjSWEET17.2 contained 5 TMHs, BjSWEET4.1 and BjSWEET2.2 contained 8 TMHs. Six proteins (BjSWEET17.3, BjSWEET4.2, BjSWEET8.1, BjSWEET8.2, BjSWEET8.3 and BjSWEET8.5) contained 6 TMHs, and the remaining BjSWEETs all contained 7 TMHs (Fig. 3). These results revealed significant variation among the 66 SWEET gene family members.

### Chromosomal localization and gene duplication of *BjSWEETs*

**Fig. 4:**
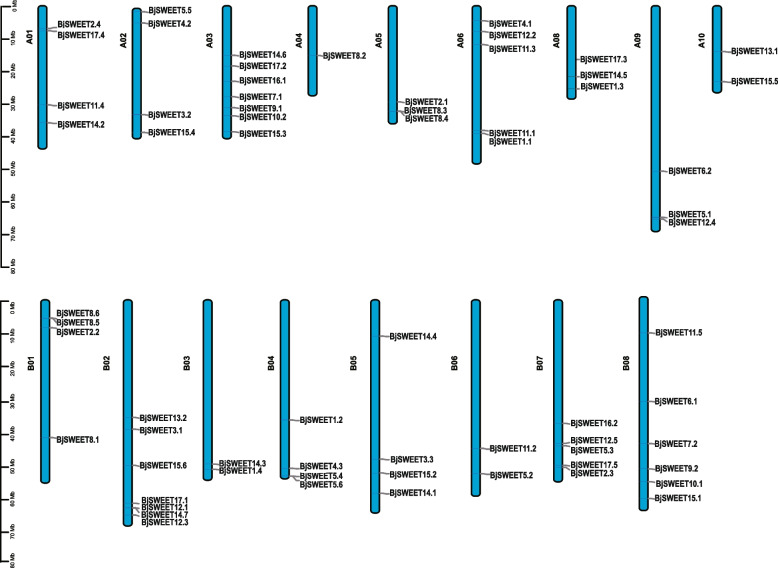
The distribution of *SWEET* genes on chromosomes in *B. juncea*. Chromosome size is indicated by its relative length. The scale bar represents megabases (Mb). The physical locations of *BjSWEETs* are drawn on each chromosome

The chromosomal distribution of *BjSWEETs* was studied according to their physical location in the *B. juncea* genome. The results exhibited that a total of 66 *SWEET* gene family members in *B. juncea* were not evenly distributed across 17 chromosomes (Fig. 4). It was found that 32 genes were located in the A subgenome, 34 were located in the B subgenome. And 7 *BjSWEET* genes were found on the A03 and B02 chromosomes, but only one *BjSWEET8.2* gene was identified on the A04 chromosome, while no *BjSWEET* gene was located on chromosome A07. Furthermore, we studied gene duplications within 66 *BjSWEET* genes by analyzing their collinearity (Fig. S3, Table S3). There were 118 pairs of segment duplicates, and some *BjSWEET* genes were repeatedly involved in the gene duplication events. Among these gene pairs, 6 pairs were found on the same chromosome and 112 pairs were distributed on diverse chromosomes, indicating that segmental duplication is the primary expansion model of SWEET gene family in *B. juncea*, and that some *BjSWEETs* were probably obtained by gene segmental duplication.

### *Cis*-elements in the promoters of *BjSWEET* genes

**Fig. 5:**
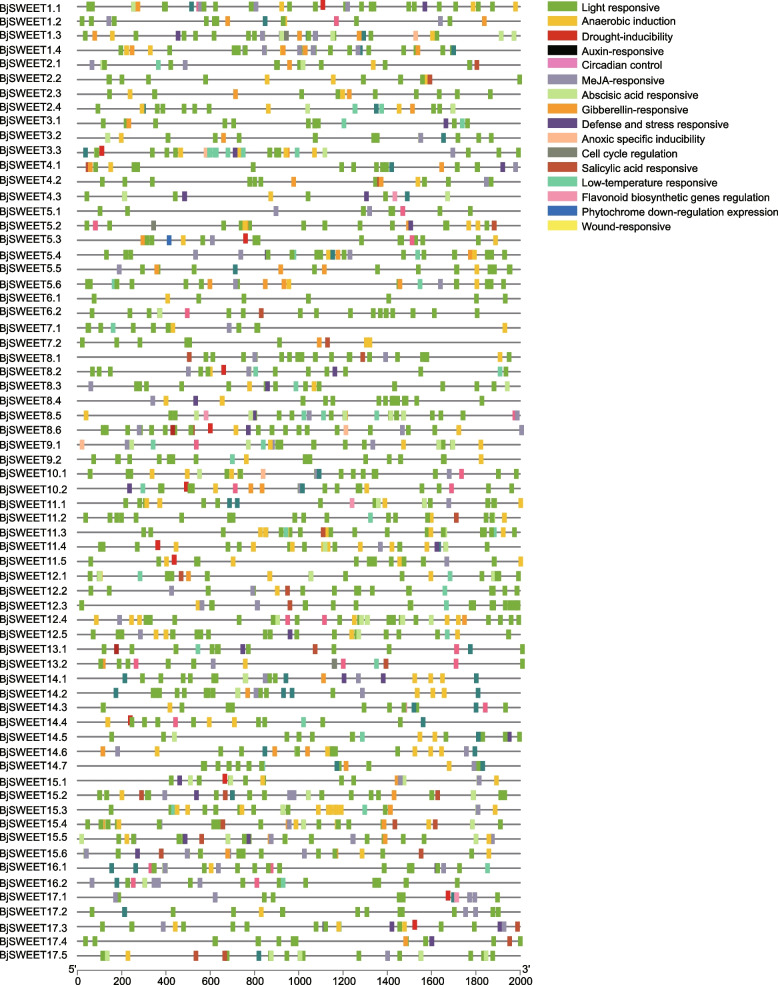
The prediction results of the *cis*-elements of the SWEET gene family in *B. juncea*. The *cis*-elements analysis was performed with the 2.0 kb upstream region using the online tool PlantCARE. Different plant hormone-related response elements, stress-responsive elements, light-responsive elements and *cis*-elements related to growth and development were showed in different colors

To further analyze the potential regulatory mechanisms of the *BjSWEET* genes, we predicted the *cis*-elements in the promoters of these genes using the online tool PlantCARE. We finally selected 20 *cis*-elements (Fig. 5, Table S4). Among them, there were 5 plant hormone-related response elements, including gibberellin (GA), auxin, abscisic acid (ABA), salicylic acid (SA) and methyl jasmonate (MeJA). And 8 stress-related response elements, including anaerobic, defense and stress response, drought, hypoxia-specific, wound, circadian control, low temperature-responsive elements, and gene regulation of flavonoid biosynthesis, were identified. Additionally, light responsive elements and *cis*-elements related to plant growth and development, such as meristem expression, endosperm expression, seed-specific regulation, and differentiation of palisade mesophyll cells were detected. Compared with other *cis*-elements, light response elements were present in the promoters of all 66 *BjSWEET* genes, implying the universal involvement of *BjSWEET* genes in light control expression. These results indicated that *BjSWEET* genes likely play essential roles in plant growth and development, abiotic and biotic stress responses, hormone regulation, and light-controlled expression.

### Expression pattern analysis of *BjSWEET* genes

**Fig. 6:**
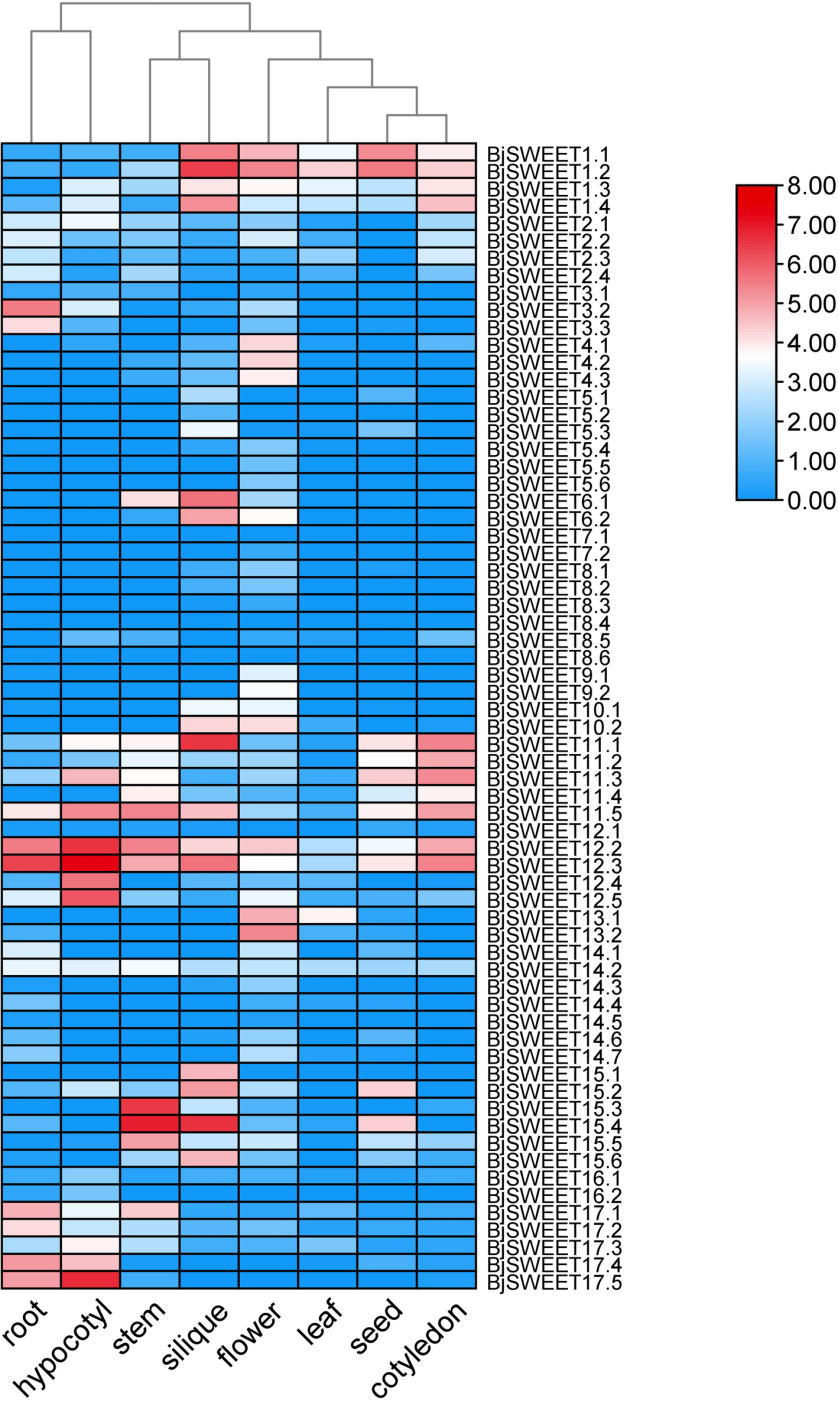
Heatmap of the expression profiles of all the *BjSWEET* genes in 8 different tissues of *B. juncea*. The expression abundance of each transcript is represented by the normalized fragments per kilobase pair per million (FPKM) value and displayed as colored boxes from green (lower expression) to red (higher expression)

Based on the transcriptome data, we constructed an expression heatmap of the *BjSWEET* genes in eight different tissues, including root, hypocotyl, stem, silique, flower, leave, seed and cotyledon (Fig. 6, Table S5). The expression patterns were further investigated to elucidate the potential biological functions of the *BjSWEET* genes. The results showed that the expression of the *BjSWEET* genes was generally low in the leaves. *BjSWEET7.1*, *BjSWEET8.6* and *BjSWEET8.4* were not expressed in eight tissues. Certain genes were specifically expressed in tissues, for example, *BjSWEET15.4* presented the highest expression in stems, but its expression was much lower in other tissues. *BjSWEET1.2* and *BjSWEET13.2* were only highly expressed in flowers. And *BjSWEET17.4* was more highly expressed in roots and hypocotyls than in other tissues. These results suggested that genes highly expressed in specific tissues may play a crucial role in the functional expression of these tissues.

### Expression profiles of* BjSWEET *genes under drought treatment via qRT-PCR

**Fig. 7:**
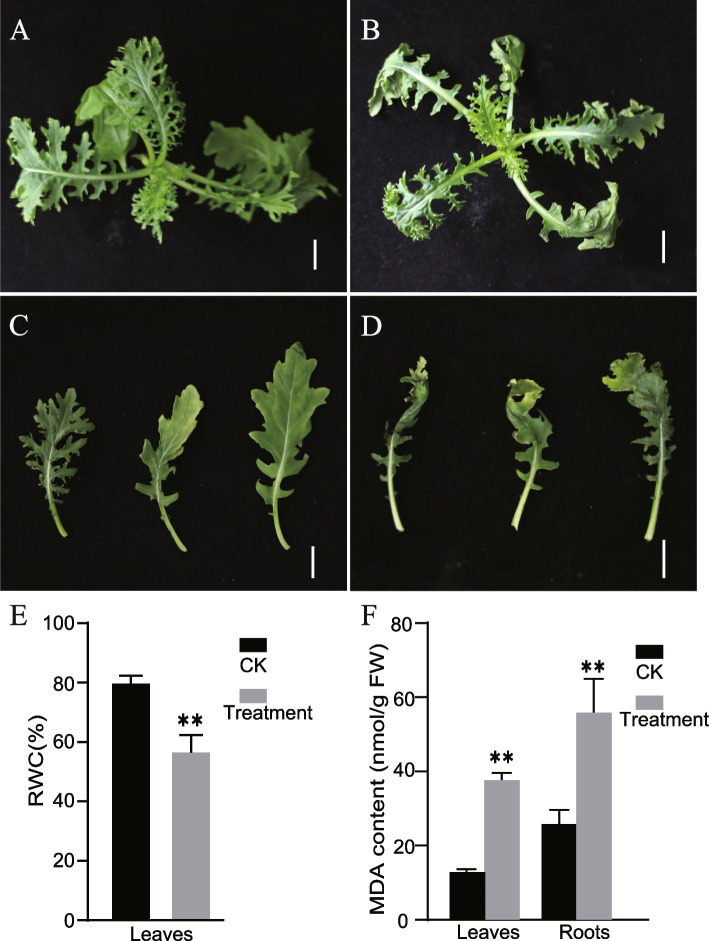
Phenotypes, physiological, and biochemical difference between mustard XC under drought stress treatment and sterile water treatment. **A** The phenotype of XC under well sterile watered condition (CK). **B** The phenotype of XC under drought stress. **C** Representative leaves of XC under well sterile watered condition (CK). **D** Representative leaves of XC under drought stress. **E** The leaf relative water content (RWD) between drought stress and sterile water treatment (CK). **F** The malondialdehyde (MDA) content in leaves and roots between drought stress treatment and sterile water treatment (CK). Bar = 1 cm

**Fig. 8:**
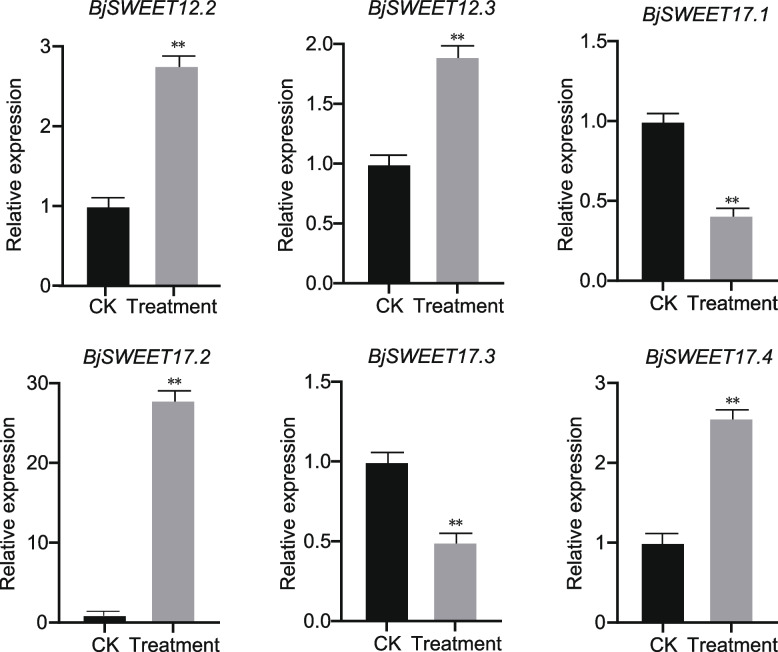
Expression analysis of *BjSWEET12* and *BjSWEET17* under drought treatment by qRT‒PCR. The data are presented as the means ± standard deviations (STDEV) (*n* = 3). Asterisks indicate significant differences between sterile watered (CK) and drought stress treatment (Student's t test, ** *p* < 0.01)

To further identify candidate *BjSWEET* genes involved in drought response, PEG6000 was used to simulate drought stress. Leaves from well-watered plants grow well (Fig. 7A, C). However, the leaves from drought treated mustard showed wilting and leaf edge damage (Fig. 7B, D). Drought stress significantly reduced the leaf relative water content when compared it with the well-watered mustard (Fig. 7E). Under drought stress, the MDA content in leaves increased significantly when compared it with the well-watered leaves (Fig. 7F). Although no significant phenotypic variation have found in the roots, the root showed an impressive capacity to influence its architecture in response to drought. The MDA content in the roots also increased significantly after drought stress treatment (Fig. 7F). The analysis of the *BjSWEETs* expression levels in eight tissues showed that *BjSWEET17.1*, *BjSWEET17.2*, *BjSWEET17.3*, *BjSWEET17.4*, *BjSWEET12.2*, and *BjSWEET12.3* were highly expressed in roots. SWEET proteins are known to participate in vacuolar sugar transport in roots to exert their drought resistance. Thus, the relative expression of these genes under drought stress was measured using qRT-PCR (Fig. 8, Table S6). The results showed that the expression profiles of the *BjSWEET* genes differed under drought stress. Compared with those in the control, the expression of *BjSWEET17.1* and *BjSWEET17.3* decreased, while the expression of *BjSWEET17.2*, *BjSWEET17.4*, *BjSWEET12.2*, *BjSWEET12.3* and *BjSWEET12.3* were significantly up-regulated under drought treatment. These results suggested that *BjSWEET17.2*, *BjSWEET17.4*, *BjSWEET12.2*, and *BjSWEET12.3* were likely involved in the response to drought stress.

## Discussion

*SWEET* genes have exhibited a widespread presence in plants, animals, and prokaryotes. They play an important role in many physiological processes, such as the regulation of metabolism, plant growth and development, plant‒pathogen interactions, and stress responses [9]. The identification and functional studies of *SWEET* genes in *Arabidopsis* [3], rice [29, 30], maize [31] and microorganisms have revealed a wide range of biological functions of *SWEET* genes in different plant species [32, 33]. The regulatory function of the SWEET gene family is variable in different species, hence, more species-specific information is required to determine these roles. In this study, 66 *BjSWEET* genes have been identified in *B. juncea*. Additionally, 34 *BrSWEET* genes and 37 *BniSWEET* genes have been identified in *B. rapa* and *B. nigra* (Table S7). Phylogenetic analysis showed that SWEETs may originate from Clade II, this is consistent with previously result that plant SWEETs have originated from Clade II [34]. Most of the *BjSWEET* genes have 2 orthologous copies. While, *BjSWEET5, BjSWEET8, and BjSWEET14* have 6 orthologous copies. Whole-genome duplication/triplication events were intertwined with segregation and rediploidization in plants, and had different effects on gene families. The expanded SWEET gene family in *B. rapa* following whole-genome triplication events showed complex regulation [25].

Eukaryotes possess multiple copies of *SWEET* genes, which can facilitate the diversification of metabolic regulation. Subcellular localization analysis showed that most of BjSWEET proteins were localized in the cell membrane. While, some BjSWEET proteins were co-localized proteins with different signal peptides. The analysis of gene structure, conserved motif, and multiple sequence alignment analysis of the *BjSWEETs* showed that 81.8% of the *BjSWEETs* had seven transmembrane helices (TMHs), and all the SWEET gene family members contained the MtN3/saliva domain, suggesting that the *BjSWEET* genes were relatively conserved during evolution. Gene structure analysis found that most of the *BjSWEETs* had six exons, which was consistent with the finding that the last common ancestor of angiosperms (LCA) had six exons. According to the analysis of transmembrane helices, approximately 81.8% of BjSWEET proteins contained seven typical transmembrane α-helical domains. The number of THMs varied in the remaining BjSWEET proteins, and these results were consistent with the finding that the number of THMs increased or decreased in MtN3/saliva/SWEET proteins [9]. The phylogenetic evolutionary analysis indicated that the *BjSWEET* genes could be classified into 4 subfamilies. The evolutionary sequence of the 4 subfamilies is as follows: Clade I, Clade II, Clade III and Clade IV [35]. The expression of genes is mostly related to the *cis*-elements in the promoter regions. *Cis*-element analysis of the promoter regions revealed that the promoters of *BjSWEET* genes contained a large number of plant hormone-related response elements, stress-related response elements, growth and development elements, and light-responsive regulatory elements, indicating the possible involvement of these genes in related functional expression in *B. juncea.* It is widely known that sugar transporters are affected by exogenous hormones. In apple, abscisic acid (ABA) signal could enhance sugar accumulation by activating the expression of MdSWEET9b [36]. *HcSWEET* genes from *Hemerocallis citrina* may response to various hormones, light, and stresses [37]. Light responsive elements were the most diverse and abundant in the promoter regions of *BjSWEET* genes, implying the universal involvement of *BjSWEET* genes in light control expression. In *Capsicum annuum*, the light responsive elements are the most abundant *cis*-elements in the promoters of *CaSWEET* genes [38].

One of the crucial functions of roots is to extract water from the soil to provide water to plants. The root shows an impressive ability to influence its architecture in response to drought [39]. In *Arabidopsis*, *AtSWEET17,* which acted as a sugar transporter, participated in the sugar transport of root vacuoles and played an important role in drought stress [8]. *AtSWEET11* and *AtSWEET12* are induced in leaves, while *AtSWEET11-15* have been upregulated in roots under drought stress [40]. *SWEET17* is mainly expressed in the vasculature and meristem cells of roots, and *SWEET12* is more highly expressed in roots. The *BjSWEET* genes may be involved in the expression of genes related to roots. The transcript expression analysis of *BjSWEETs* genes in eight tissues showed that *BjSWEET17.1*, *BjSWEET17.2*, *BjSWEET17.3, BjSWEET17.4*, *BjSWEET12.2*, and *BjSWEET12.3* were highly expressed in roots, and some *SWEET* genes in *B. juncea* were specifically expressed in flowers, roots and stems, indicating that these genes play a role in reproductive processes and metabolic regulation in roots and stems. However, apart from light responsive elements, MeJA-responsive, abscisic acid responsive, salicylic acid responsive, and defense and stress responsive elements have been found in the promoters of *BjSWEET* genes. To further understand whether the *BjSWEET12* and *BjSWEET17* are involved in drought resistance, this study analyzed the expression profiles of *SWEET12* and *SWEET17* under drought treatment. The results showed that the expression of *SWEET17.2*, *SWEET17.4*, *SWEET12.2* and *SWEET12.3* were significantly up-regulated under drought treatment, suggesting that these genes were likely involved in the response to drought stress. These results lay a solid foundation for further study of the related molecular functions and mechanisms of *SWEET* genes in *B. juncea*.

## Materials and methods

### Identification and bioinformatics analysis of *BjSWEET* genes

*Arabidopsis* genome annotation information and SWEET protein sequences were obtained from TAIR (www.arabidopsis.org). Based on the homology of the *Arabidopsis SWEET* genes, using the AtSWEET protein sequence as the query sequence, we originally obtained the *BjSWEET* genes from the *B. juncea* ‘Xuecai’ (XC) genome (PRJCA015688) from the National Genomics Data Center (https://ngdc.cncb.ac.cn/gwh). The *SWEET* genes from *B. rapa* genome (Brara_Chiifu_V4.1/) and *B. nigra* genome (Brani_Ni100_V2/) were obtained from BRAD (http://brassicadb.cn) by the BLAST QUI Wrapper (Two Sequences Sets) function of TBtools [41], with an E-value threshold of 1e-5. Then we used the Venn and UpSet plot functions of TBtools to deduplicate these *SWEET* genes with minimum overlap size is 1. Interpro (www.ebi.ac.uk/interpro/) was used to screen the conserved domains (PF03083), and the SWEET gene family members were ultimately obtained from *B. juncea*. The Expasy tool (https://web.expasy.org/protparam/) was utilized to predict and analyze a variety of parameters of BjSWEET proteins, including encoded amino acids, relative molecular mass, and theoretical isoelectric points. Additionally, we predicted the subcellular localization of the SWEET protein in *B. juncea* using the online tool Plant-mPLoc (www.csbio.sjtu.edu.cn/bioinf/plant-multi/).

### Phylogenetic and multiple sequence alignment analysis of SWEET sequences in *B. juncea* and Arabidopsis

The MAFFT tool was used to compare SWEET protein sequences from *A.thaliana*, *B. rapa*, *B. nigra*, and *B. juncea* with 1,000 bootstrap replicates [42]. Additionally, the phylogenetic tree of SWEET protein was constructed using IQ-TREE [43], employing the maximum-likelihood (ML) approach with 1000 replications with the parameters –m, TEST, -redo, -n, 1000.

### Gene structure, conserved motifs and transmembrane helices of BjSWEETs

The SWEET protein sequences were submitted to the One Step Build a ML Tree function of TBtools to determine phylogenetic relationships. Using the Simple MEME wrapper function to predict the conserved motifs of the BjSWEET proteins with the maximum number of motifs 10, and e-value 10. The conserved domain information of the BjSWEET proteins was analyzed using the online tool NCBI Batch CD-search (www.ncbi.nlm.nih.gov). Then, the *B. juncea* SWEET genome annotation files, phylogenetic relationship information, conserved motif information, and domain information were submitted to the Gene Structure View function for visualization. The FASTA files of the BjSWEET protein sequences were submitted to the online tool Tmhmm 2.0 Service (https://services.healthtech.dtu.dk/services/TMHMM-2.0/) to obtain transmembrane helix structure information.

### Chromosomal localization and gene duplication analysis of *BjSWEETs*

The genome annotation files and IDs of the *BjSWEET* genes were obtained from the sequenced *B. juncea* genome. The gene location visualize function of the GFF/GTF function of TBtools was used to visualize the chromosomal location of SWEET gene family members in *B. juncea*. The collinearity analysis of the *SWEET* gene family members in *B. juncea* was performed using the MCScanX function of TBtools with an E-value threshold of 1e-10.

### *Cis*-elements in the promoters of *BjSWEET* genes

We obtained the 2000 bp upstream of the promoters of *BjSWEET*s by Gtf/Gff3 sequences extraction and fasta extraction function of TBtools. The online tool PlantCARE (https://bioinformatics.psb.ugent.be/webtools/plantcare/html/) was used to predict the promoter *cis*-elements. Then, we performed data screening based on the obtained Tab file and displayed it by the gene structure view function of TBtools selected with “Fill in gradient mode” option.

### Gene expression pattern analysis of *BjSWEETs*

The *SWEET* gene expression data for eight different tissues were derived from our previous study, and the XC genome was used as a reference [44]. Fragments per kilobase pair of transcript per million mapped reads (FPKM) values of these *BjSWEET* genes across 8 different tissues have been listed in Table S5. The heatmap function of TBtools was subsequently used to visualize the expression of *BjSWEET* genes in root, hypocotyl, stem, silique, flower, leave, seed and cotyledon.

### Drought treatment

The potherb mustard ‘Xuecai’ (XC) is widely used as a fresh and a pickled vegetable [44]. The selfed seeds of *B. juncea* inbred line XC used in this study were harvested from the experimental field at Xinyang Normal University (Xinyang, China). The seed of *B. juncea* inbred line XC was used in this study. *Brassica juncea* XC was planted in 1/2 MS agar at 25°C with 16 h of light and 8 h of darkness for 2 weeks. Then, 2 ml of sterile water (CK) or 10% (w/v) PEG6000 was injected evenly into the culture medium near the roots of each plant, respectively. Each group had 16 mustard plants for replication. The mustard XC plants treated with sterile water (CK) were used as the control plants. For phenotypic observation, samples were photographed after 7 days of treatment. Then, we sampled the roots and leaves of the plants in the CK and PEG6000 treatment.

### Measurement of relative water content and malondialdehyde (MDA) content

The leaf relative water content (RWC) was measured according to a previously described method [45]. Mustard leaves with well-watered and drought treatment were collected to record the fresh weight (FW). Then, the dry weight (DW) was determined after the leaves were dried at 105°C for 2 h and 80°C until constant weight. The RWC was calculated with the formula: RWC (%) = (FW-DW)/(TW-DW) × 100%. The malondialdehyde (MDA) content was further examined with the thiobarbituric acid method [46]. The samples for RWC and MDA estimation were performed with 3 biological replicates. And 4 mustard plants were used for each replicate.

### RNA extraction and qRT‒PCR

Total RNA was extracted with a TaKaRa MiniBEST Plant RNA Extraction Kit, and a RevertAid First Strand cDNA Synthesis Kit was used for cDNA synthesis. Quantitative fluorescence primers were designed using the batch qRT**‒**PCR primer design function and the primer check function of TBtools to check primers specificity. Based on the general principles of primer designation, for example the length of the primer is approximately 20 bp, the Tm is between 58 and 60 degrees Celsius, and the GC content is 40–60% as appropriate, to determine the final primer sequence (Table S8). The transcript expression levels of *BjSWEET* genes were quantified by qRT–PCR [47], and analyzed by using the 2^−ΔΔCT^ method [48]. The *BjActin* gene was used as the internal control to normalize the transcript levels [49]. Each sample was performed with three technical replicates. Each reaction (total volume of 20 μL) consisted of 8.4 μL of diluted cDNA, 0.8 μL of forward primer, 0.8 μL of reverse primer, and 10 μL of 2 × SYBR qRT‒PCR Mix. The PCR cycle conditions were as follows: 95℃ for 2 min, 95℃ for 15 s, and 60℃ for 30 s. After return to the second step, 40 cycles were performed, a melting curve was generated to obtain the qRT‒PCR data. Then, the gene expression data was processed by IBM SPSS Statistics version 22.0. The Shapiro–Wilk test was used to evaluate whether the variables gene expression data between different groups were normally distributed. The gene expression data obtained by qRT‒PCR were presented as the means ± STDEV (standard deviation) and visualized using GraphPad Prism 8 software. The Student's t test was used to compare the significant difference between transcript expression levels of *BjSWEET* genes between drought stress and sterile water treatment. Asterisk denotes statistically significant differences between sterile watered (CK) and drought stress treatment (Student's t test, ** *p* < 0.01).

## Supplementary Information

Additional file 1: Figure S1. Multiple sequence alignment of SWEET family proteins in Arabidopsis and B. juncea. The conserved domain of the BjSWEET proteins was localized at the N-terminus. The box showed that all 66 BjSWEET genes have a segment of conserved sequence constituting the conserved MtN3-slv or PQ-loop superfamily.

Additional file 2: Figure S2. Specific-conserved motifs of SWEET family proteins in B. juncea. The colored boxes represent different conserved motifs with different sequences and sizes.

Additional file 3: Figure S3. Gene duplication of BjSWEETs on chromosomes. Colored lines indicate duplicated SWEET gene pairs on the same chromosome.

Additional file 4: Table S1. Characteristics of the SWEET genes identified in B. juncea. Table S2. Conserved motifs and functional annotation of BjSWEETs. Table S3. The gene duplication pairs of SWEET genes in B. juncea. Table S4 Analysis of cis-acting elements in the SWEET gene family of B. juncea. Table S5 FPKM values of BjSWEET genes in 8 different tissues of B. juncea. Table S6 BjSWEET genes analyzed by qRT‒PCR. Table S7 SWEET genes in B. nigra and B. rapa. Table S8 Primers used for qRT‒PCR.

## Data Availability

The transcriptome data was deposited in National Genomics Data Center with accession number PRJCA015688. Other datasets used and analyzed during the current study are contained within the article or supplementary files.
