# Supplementary material for: Genome wide identification of BjSWEET gene family and drought response analysis of BjSWEET12 and BjSWEET17 genes in Brassica juncea
Source: BMC Plant Biol. 2024 Nov 19;24:1094. doi: 10.1186/s12870-024-05815-w (PMC11575039; doi:10.1186/s12870-024-05815-w)
Supplement: Supplementary file 1 — Additional file 1: Figure S1. Multiple sequence alignment of SWEET family proteins in Arabidopsis and B. juncea. The conserved domain of the BjSWEET proteins was localized at the N-terminus. The box showed that all 66 BjSWEET genes have a segment of conserved sequence constituting the conserved MtN3-slv or PQ-loop superfamily. [file 12870_2024_5815_MOESM1_ESM.pdf]

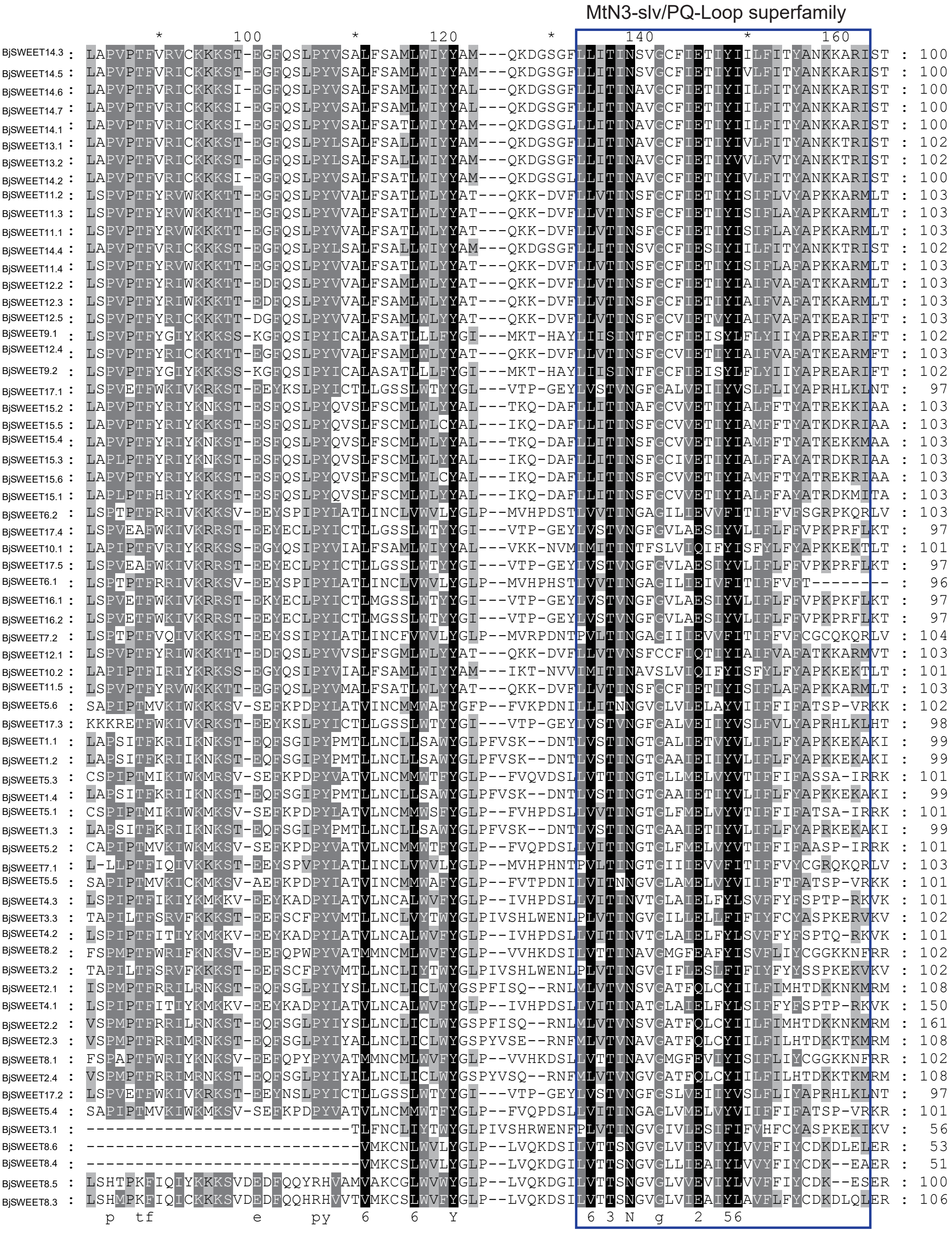
MtN3-slv/PQ-Loop superfamily
100
120
140
160
