# Supplementary figures and images for: Genome wide identification of BjSWEET gene family and drought response analysis of BjSWEET12 and BjSWEET17 genes in Brassica juncea
Source: BMC Plant Biol. 2024 Nov 19;24:1094. doi: 10.1186/s12870-024-05815-w (PMC11575039; doi:10.1186/s12870-024-05815-w)

Motif4

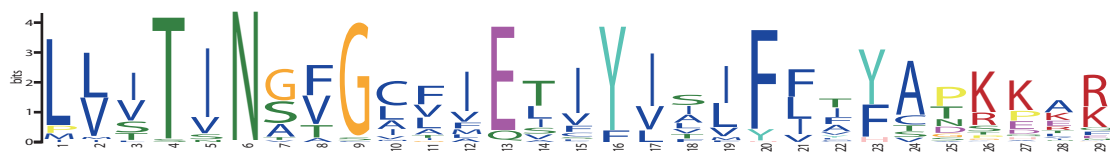

Motif5

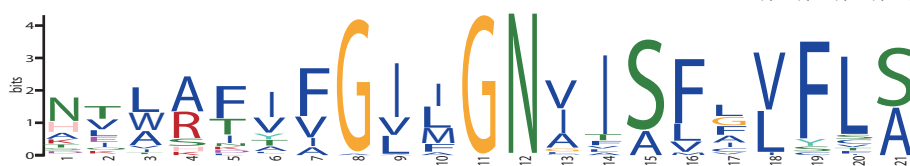

Motif9

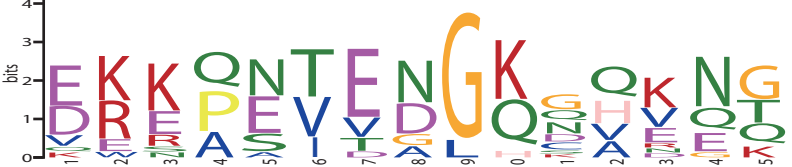

Motif10

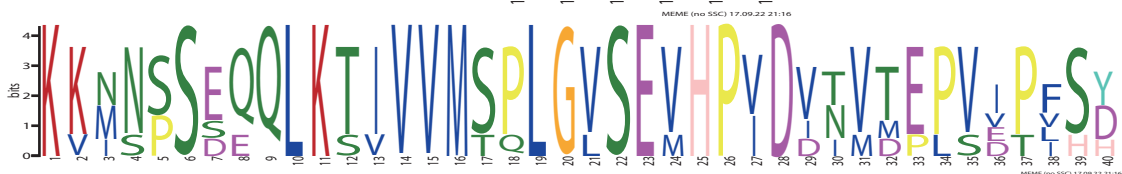

MEME (no SSC) 17.09.22 21:16

MEME (no SSC) 17.09.22 21:16

Supplement: Supplementary file 2 — Additional file 2: Figure S2. Specific-conserved motifs of SWEET family proteins in B. juncea. The colored boxes represent different conserved motifs with different sequences and sizes. [file 12870_2024_5815_MOESM2_ESM.pdf]

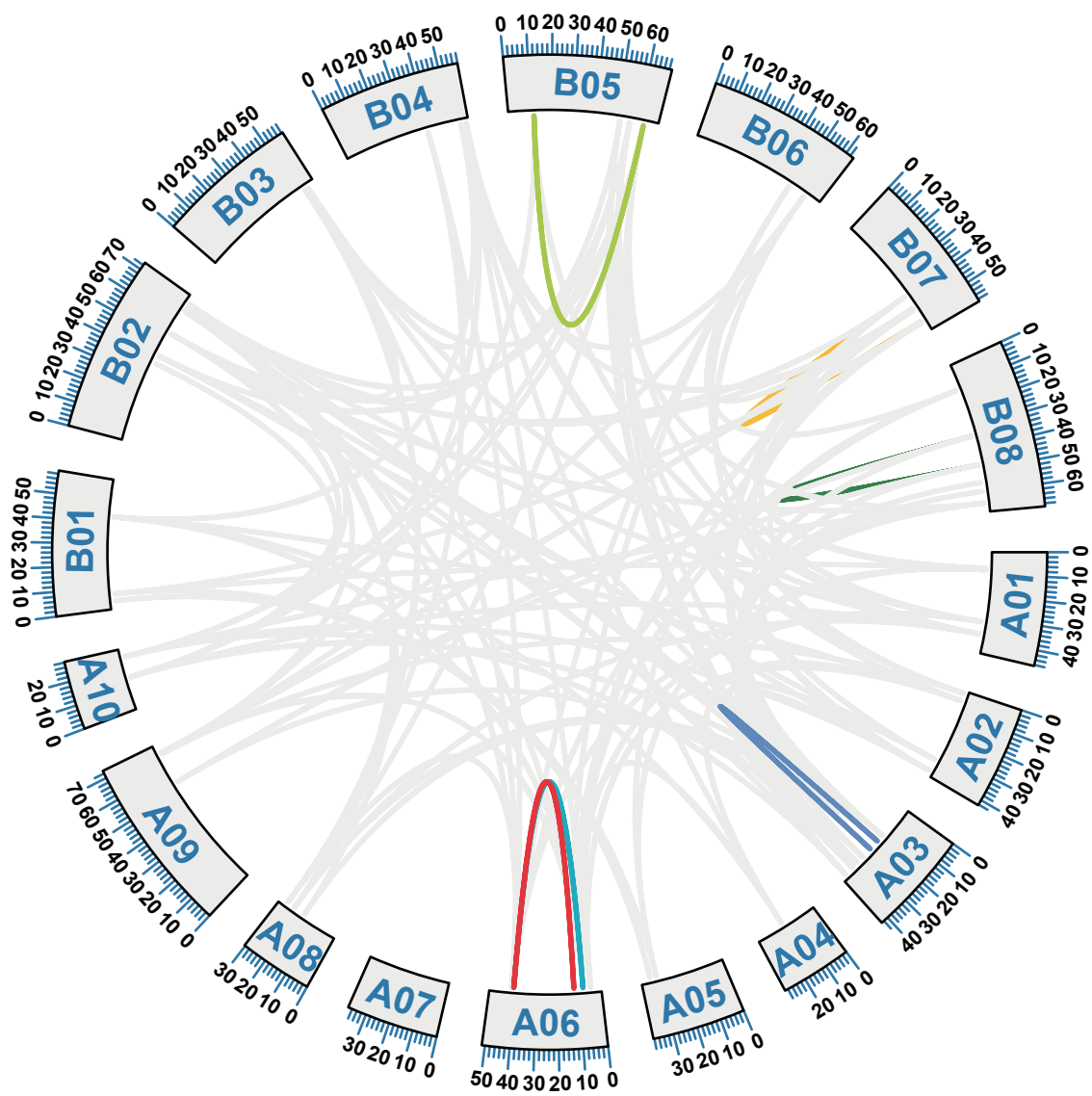

Supplement: Supplementary file 3 — Additional file 3: Figure S3. Gene duplication of BjSWEETs on chromosomes. Colored lines indicate duplicated SWEET gene pairs on the same chromosome. [file 12870_2024_5815_MOESM3_ESM.pdf]
